# Synthesis of multi-organo-functionalized fibrous silica KCC-1 for highly efficient adsorption of acid fuchsine and acid orange II from aqueous solution

**DOI:** 10.1038/s41598-021-81080-3

**Published:** 2021-02-01

**Authors:** Roozbeh Soltani, Rasool Pelalak, Mahboubeh Pishnamazi, Azam Marjani, Ahmad B. Albadarin, Shaheen M. Sarkar, Saeed Shirazian

**Affiliations:** 1 Department of Chemistry, Islamic Azad University, Arak Branch, Arak, Iran; 2 Institute of Research and Development, Duy Tan University, Da Nang, 550000 Vietnam; 3 Faculty of Environmental and Chemical Engineering, Duy Tan University, Da Nang, 550000 Vietnam; 4 Faculty of Pharmacy, Duy Tan University, Da Nang, 550000 Vietnam; 5 Department for Management of Science and Technology Development, Ton Duc Thang University, Ho Chi Minh City, Vietnam; 6 Faculty of Applied Sciences, Ton Duc Thang University, Ho Chi Minh City, Vietnam; 7 Department of Chemical Sciences, Bernal Institute, University of Limerick, Limerick, Ireland; 8 Laboratory of Computational Modeling of Drugs, South Ural State University, 76 Lenin Prospekt, Chelyabinsk, Russia 454080

**Keywords:** Chemistry, Materials science, Nanoscience and technology

## Abstract

Multi-functionalized fibrous silica KCC-1 (MF-KCC-1) bearing amine, tetrasulfide, and thiol groups was synthesized via a post-functionalization method and fully characterized by several methods such as FTIR, FESEM, EDX-Mapping, TEM, and N_2_ adsorption–desorption techniques. Due to abundant surface functional groups, accessible active adsorption sites, high surface area (572 m^2^ g^−1^), large pore volume (0.98 cm^3^ g^−1^), and unique fibrous structure, mesoporous MF-KCC-1 was used as a potential adsorbent for the uptake of acid fuchsine (AF) and acid orange II (AO) from water. Different adsorption factors such as pH of the dye solution, the amount of adsorbent, initial dye concentration, and contact time, affecting the uptake process were optimized and isotherm and kinetic studies were conducted to find the possible mechanism involved in the process. For both AF and AO dyes, the Langmuir isotherm model and the PFO kinetic model show the most agreement with the experimental data. According to the Langmuir isotherm, the calculated maximum adsorption capacity for AF and AO were found to be 574.5 mg g^−1^ and 605.9 mg g^−1^, respectively, surpassing most adsorption capacities reported until now which is indicative of the high potential of mesoporous MF-KCC-1 as an adsorbent for removal applications.

## Introduction

Synthetic dyes are extensively used in various industries such as cosmetics, pharmaceutical, plastics, rubber, leather, textile, paper, and food, especially in developing countries and the Third World. The industrial effluents containing dyes which are produced are mainly discharged into surface waters such as rivers, ponds, and lakes^1,2^. The presence of synthetic dyes in wastewater, even at very low concentration (less than 1 mg L^−1^ in some cases), is not only aesthetically unpleasant but also causes problems for aquatic life because colored water reducing the transparency and thus blocking the penetration of sunlight into the water and consequently disrupting the process of photosynthesis^3–5^. In addition to the negative effects mentioned above, some industrial synthetic dyes are thought to be carcinogenic, mutagenic, and teratogenic in animals and human beings^6^. For instance, acid fuchsin (AF) and acid orange II (AO) are two toxic and hazardous industrial synthetic dyes that are widely used as a corrosion inhibitor and laboratory reagent in addition to from their wide usage in hair dye, wool, silk, leather, nylon, and dying textile industries^7,8^. Accordingly, the removal of such hazardous dyes from aqueous environments and waste effluents is of concern from a human health point of view.

To date, several removal methods including biosorption and adsorption^5,9,10^, ion-exchange^11,12^, catalytic degradation^13–15^, and membrane separation^16,17^ have been studied and used to remove synthetic dyes from the aqueous environments. Among them, the adsorption technique has attracted a great deal of attention because it is a more efficient, simple, versatile, cost-effective, and best-suited process for the removal of synthetic dyes like AF and AO^9,18–20^.

A vast variety of materials, including mesoporous silica materials (MSMs)^20,21^, layered doubele hydroxides (LDHs)^22^, metal–organic frameworks and their composites^18,23^, covalent organic frameworks and their composites^24–26^, Fe_2_O_3_ nanoparticles^28,29^, single-and multi-walled carbon nanotubes^30,31^, and graphene and graphene oxide-based materials^32,33^ have been utilized as an adsorbent to separate or remove the organic molecules like hydrocarbons, drugs and dyes. Among those adsorbents, MSMs-based adsorbents have shown excellent performance due to their environment-friendly water-based synthesis methods (sol–gel process), porous structure, large surface area, high pore volume, designable structure and morphology, functionalizable surface, good chemical and thermal stability, and reusability^19–21,34^. Although pure silicas are inherently able to adsorb pollutants like heavy metals and organic synthetic dyes due to their large number of surface silanol groups (through hydrogen bonding)^35,36^, it is believed that the surface functionalization process is an indispensable operation for increasing the adsorption performance of these materials^21,37,38^. For this purpose, one of the best options available for surface functionalization is the use of silane coupling agents (SCAs) which in addition to establishing a strong covalent bond with surface silanol groups possess a high diversity of organic functional groups bearing carbon, oxygen, nitrogen, and sulfur atoms^39,40^.

One of the newest members of the MSMs family is fibrous silica KCC-1, which was first synthesized and characterized in 2010 by Polshettiwar et al.^41^. Unlike conventional ordered MSMs such as FDU-12, KIT-6, KIT-5, SBA-16, SBA-15, MCM-48, and MCM-41, in which the large surface area is related to their regular pore structure^25,42^, in KCC-1 the high surface area is due to the presence of regular and concentric fibers that have grown radially from the center of the silica spheres to the outside of the sphere^43,44^. It has been reported that due to this unique feature, easier access to the surface silanols of these fibers is possible because unlike ordered MSMs, the pore-blocking phenomenon does not occur during the surface modification or synthesis process which makes them inaccessible^45–48^. Therefore, KCC-1 can be a suitable candidate for adsorption and catalysis applications where the need for high surface area and accessible active sites are the first priority. In the application of adsorption, this material can especially play the role of adsorbent via functionalization of its surface with a wide variety of functional groups as potential adsorption sites^44,47^.

In this research work, a multi-functionalized KCC-1 (MF-KCC-1) bearing amine (–NH_2_), thiol (–SH), and tetrasulfide (–S–S–S–S–) groups was synthesized via a post-modification (or post-functionalization) method and used as a potential adsorbent for removal of AF and AO dyes from water. The impact of important adsorption factors, including pH, amount of the adsorbent, initial dye concentration, and contact time, on the adsorption procedure were studied and optimal adsorption conditions were found. In order to find possible adsorption mechanisms involved in the removal process, isotherm and kinetic studies were conducted and the corresponding adsorption parameters were investigated. The adsorption performance of MF-KCC-1 was also compared with that of previous adsorbents toward AF and AO.

## Results and discussion

### Adsorbent design and synthesis strategy

**Figure 1:**
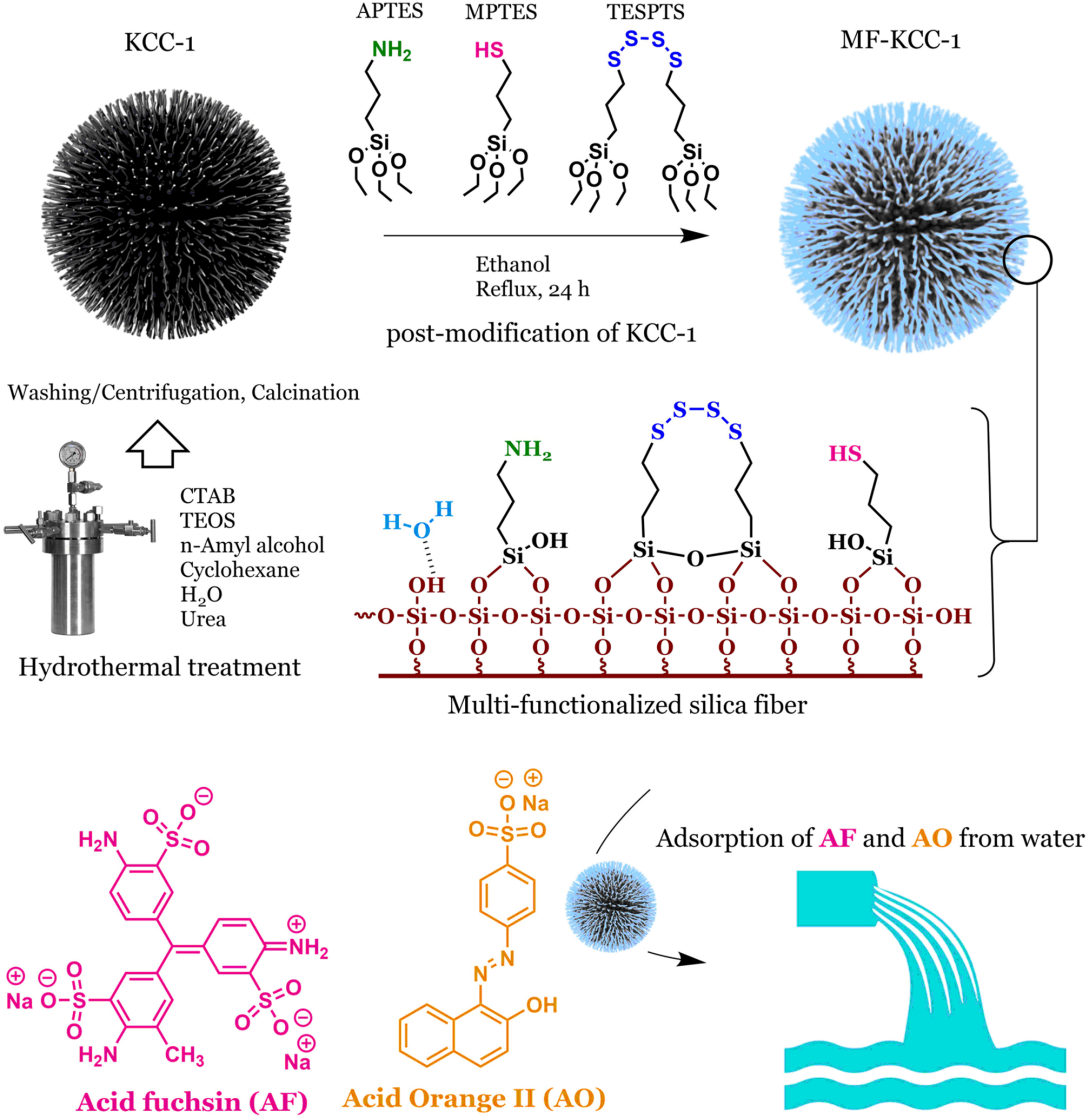
The overall process of synthesizing KCC-1 and MF-KCC-1 and molecular structure of AF and AO dyes.

Mesoporous KCC-1 was synthesized via a conventional sol–gel-hydrothermal method in a stainless-steel autoclave. In this synthesis process, TEOS, CTAB, *n*-amyl alcohol, cyclohexane, and urea were used as silica source, structure-directing agent (template), co-surfactant (for stabilizing the micelles/microemulsion droplets), co-solvent, and hydrolyzing agent, respectively. Due to the presence of many silanol groups on the surface of silica fibers of KCC-1, SCAs can be easily attached to the silica fibers and cover the entire length of the fibers by establishing strong chemical bonds as shown in Fig. 1. Unlike common ordered MSMs which are prone to pore blocking phenomenon leading essentially to the inaccessibility of a number of adsorption sites inside the pores and channels, MF-KCC-1 is able to provide more available adsorption sites to adsorbed species due to its unique fibrous structure. Also, in comparison to MF-KCC-1, the continuous channel structure in ordered MSMs limits the rate of penetration of adsorbates into these channels and the amount of adsorbate reaching adsorption sites. Therefore, it seems that the fibrous structure of KCC-1 with its high accessible surface area makes it an ideal platform for surface functionalization and use to adsorb species.

### Characterization of the KCC-1 and MF-KCC-1

#### FTIR analysis

**Figure 2:**
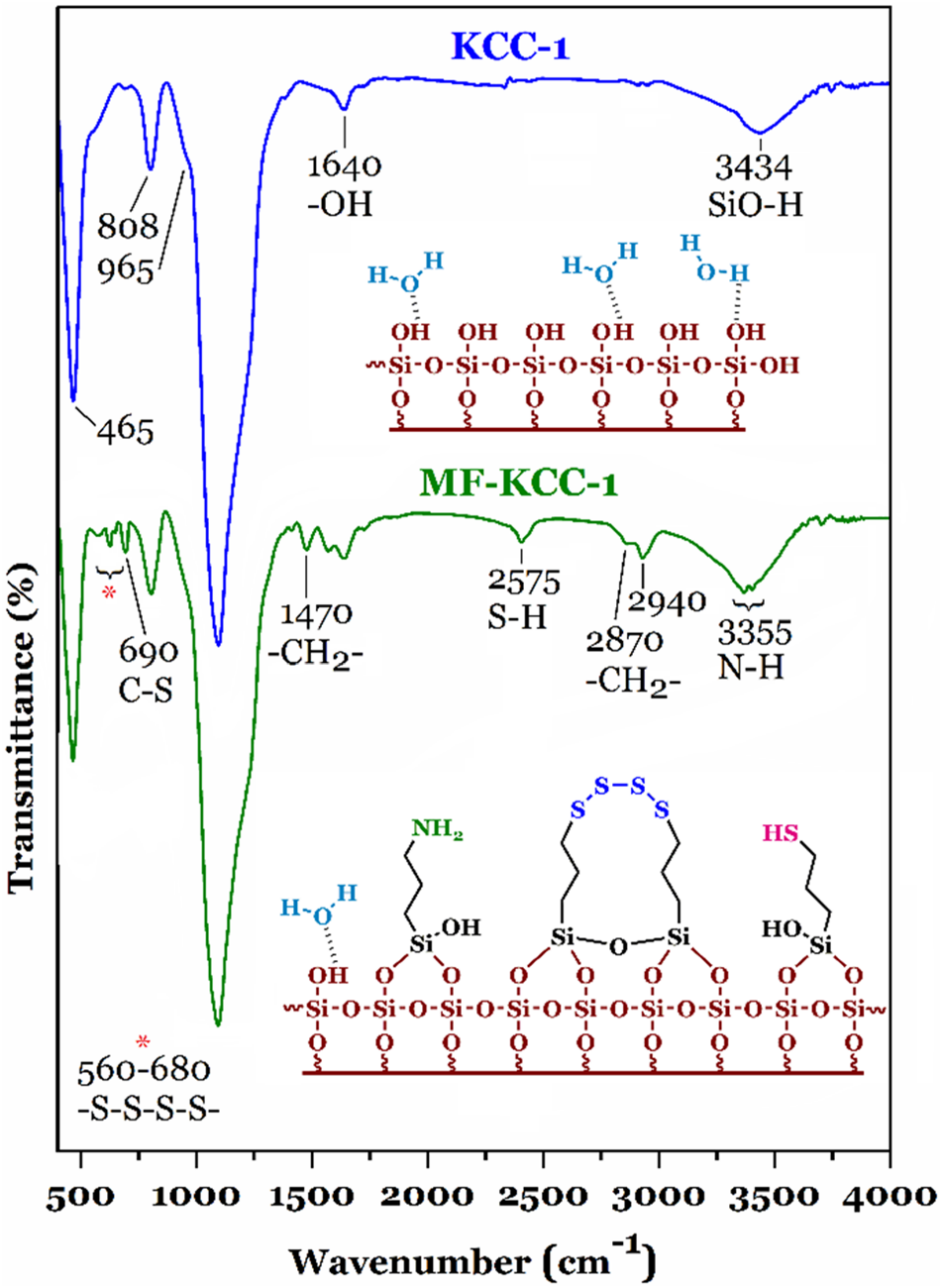
FTIR spectra of the samples.

FTIR spectra of KCC-1 and MF-KCC-1 are shown in Fig. 2. In the case of KCC-1, the characteristic absorption bands at 465 cm^−1^, 808 cm^−1^, 965 cm^−1^, and 1095 cm^−1^ are observed which are attributed to the bending vibration of Si–O–Si, Si–O stretching vibrations, Si–OH stretching vibrations, and Si–O–Si stretching vibrations, respectively. The FTIR band at 1640 cm^−1^ is due to the bending mode of surface adsorbed water molecules. The broad absorption band centered at 3434 cm^−1^ is assigned to the stretching vibrations of SiO–H groups (silanols) and O–H vibrations of surface adsorbed water molecules. The incorporation of SCAs containing –NH_2_, –SH, and –S–S–S–S– groups in the silica frameworks of KCC-1 can be qualitatively confirmed by the FTIR spectrum shown in Fig. 2. Two weak absorption bands at 560–680 cm^−1^ are seen which are due to –S–S–S–S– stretching vibrations, and C–S stretching mode at 690 cm^−1^ is observed, indicating the existence of tetrasulfide groups as well as thiol groups. FTIR band at 1470 cm^−1^ is assigned to the bending vibration of –CH_2_– groups in the SCAs structure. The weak absorption band at 2575 cm^−1^ is attributed to the S–H stretching vibration. Two characteristic absorption bands at 2870 cm^−1^ and 2940 cm^−1^ are related to the symmetric and asymmetric C–H stretching vibrations. The broad absorption band between 3600 and 3100 cm^−1^ is due to the presence of amine and hydroxyl (silanols) groups in the MF-KKC-1. The symmetric and asymmetric N–H stretching around 3355 cm^−1^ confirmed the presence of primary amine (-NH_2_) in the MF-KKC-1.

Theses above-mentioned results indicate the successful grafting of SCAs onto the KCC-1 surface and are in good agreement with the previous reports^19,21,49–51^ concerning the synthesis of silica frameworks and grafting of SCAs on nanoporous silica materials.

#### TEM, FESEM, and EDX dot mapping analyses

**Figure 3:**
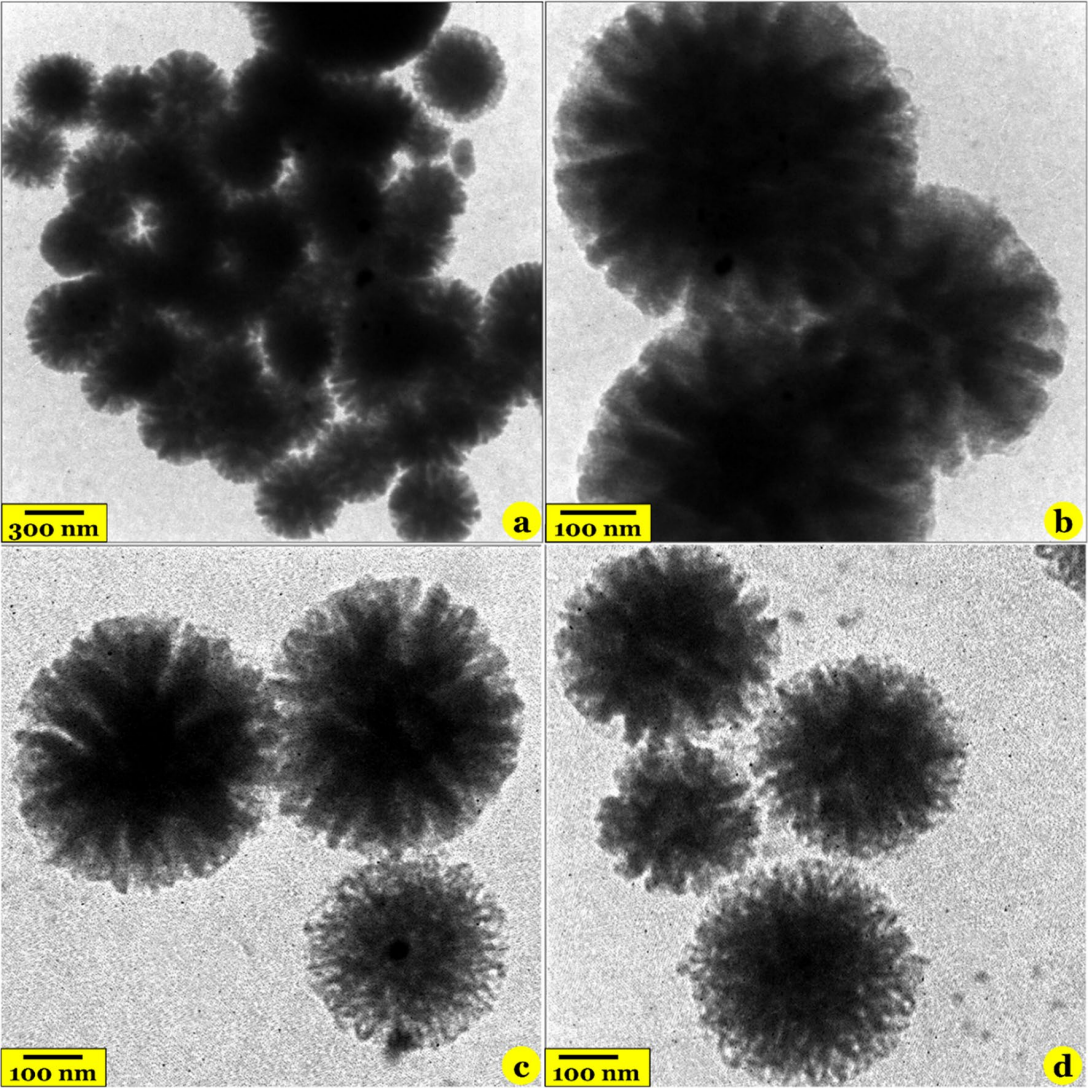
TEM images of the pure KCC-1 (**a** and **b**) and MF-KCC-1 (**c** and **d**).

**Figure 4:**
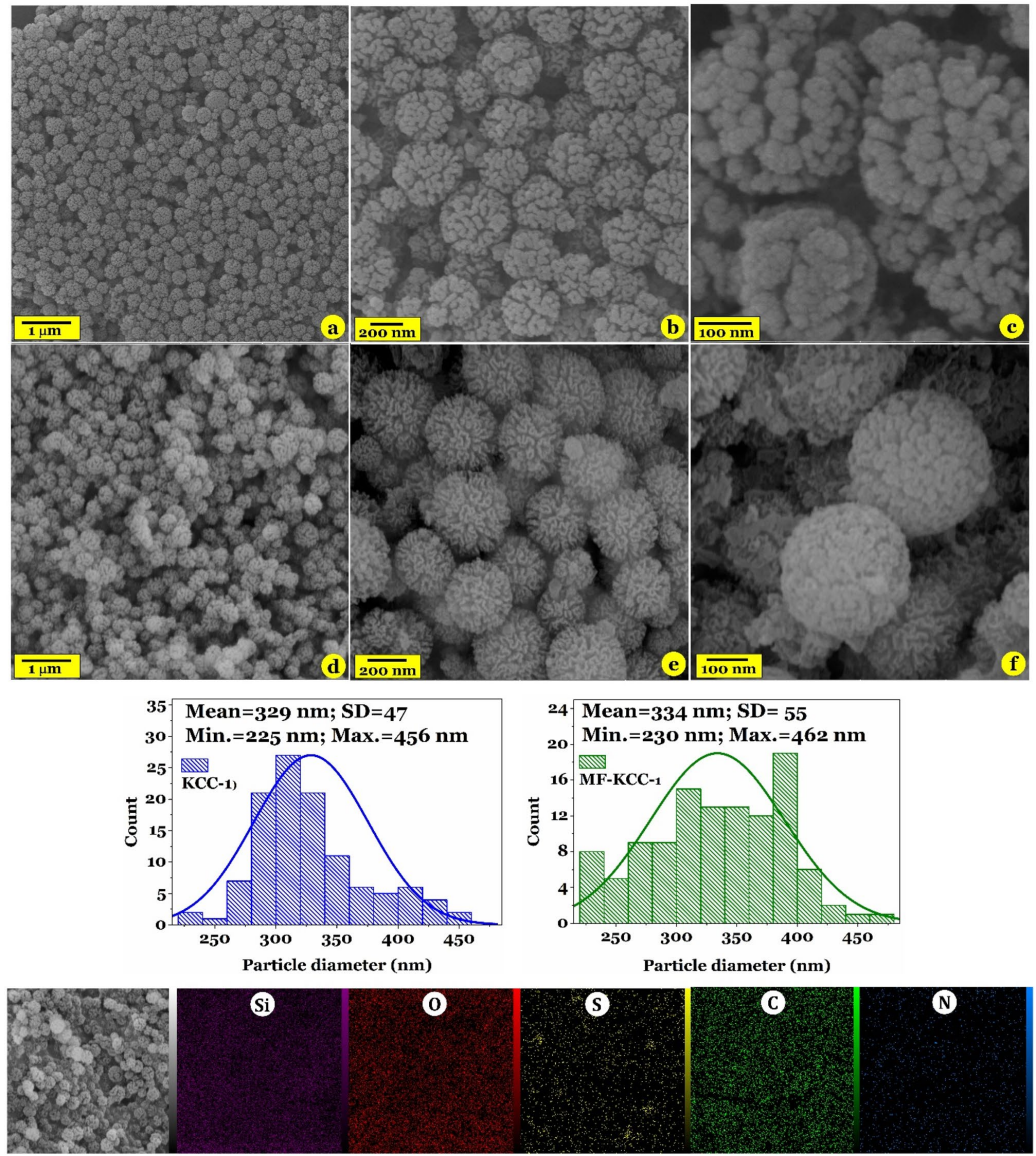
FESEM images of pure KCC-1 (**a**–**c**) and MF-KCC-1 (**d**–**f**) and corresponding particle size histogram (the third row). The fourth row indicates EDX dot elemental mapping images of the MF-KCC-1.

The fibrous structure of silica spheres (Fig. 3), the surface morphology of the samples (Fig. 4, the first and second rows), and distribution of elements on the surface of the MF-KCC-1 (Fig. 4, the third row) were observed by TEM, FESEM, and EDX mapping images, respectively.

Close inspection of KCC-1 and MF-KCC-1 by TEM technique indicate that both of these samples have dendrimeric fibers arranged in 3D space to form uniform spheres (Fig. 3). However, by comparing the TEM images of the samples, it can be seen that the density of fibers in the pure KCC-1 (Fig. 3a and b) is more than that of the MF-KCC-1 (Fig. 3c and d) which is due to the repulsion between silica fibers coated with SCAs. Similar observations have been reported by Soltani and his colleagues^20,46,47^. Consistent with previous reports, FSEM images of both KCC-1 (Fig. 4a–c) and MF-KCC-1 (Fig. 4d–f) reveal that these materials consist of colloidal spheres of uniform size with wrinkled morphology. The particle-size histograms of samples (Fig. 4, the third row) showed that the diameter of KCC-1 and MF-KCC-1 range from 225 to 456 nm and from 230 to 462 nm, respectively. The slight increase in the size of the spheres after surface modification is probably due to the chemical grafting of SCAs on the KCC-1 particles. Comparing the FESEM images of the samples, it seems that the thickness of the wrinkled layers of silica spheres has decreased after surface functionalization, which is probably due to the repulsion between the organic chains of SCAs, as mentioned above, as well as the ultrasonication process. The elemental composition of the MF-KCC-1 was obtained from EDX mapping analysis and presented in Fig. 4 (the fourth row) and reveals that MF-KCC-1 contains Si, S, O, N, and C elements which are homogeneously distributed on the surface of fibrous spheres.

#### Surface area, pore volume, and pore diameter measurements

**Figure 5:**
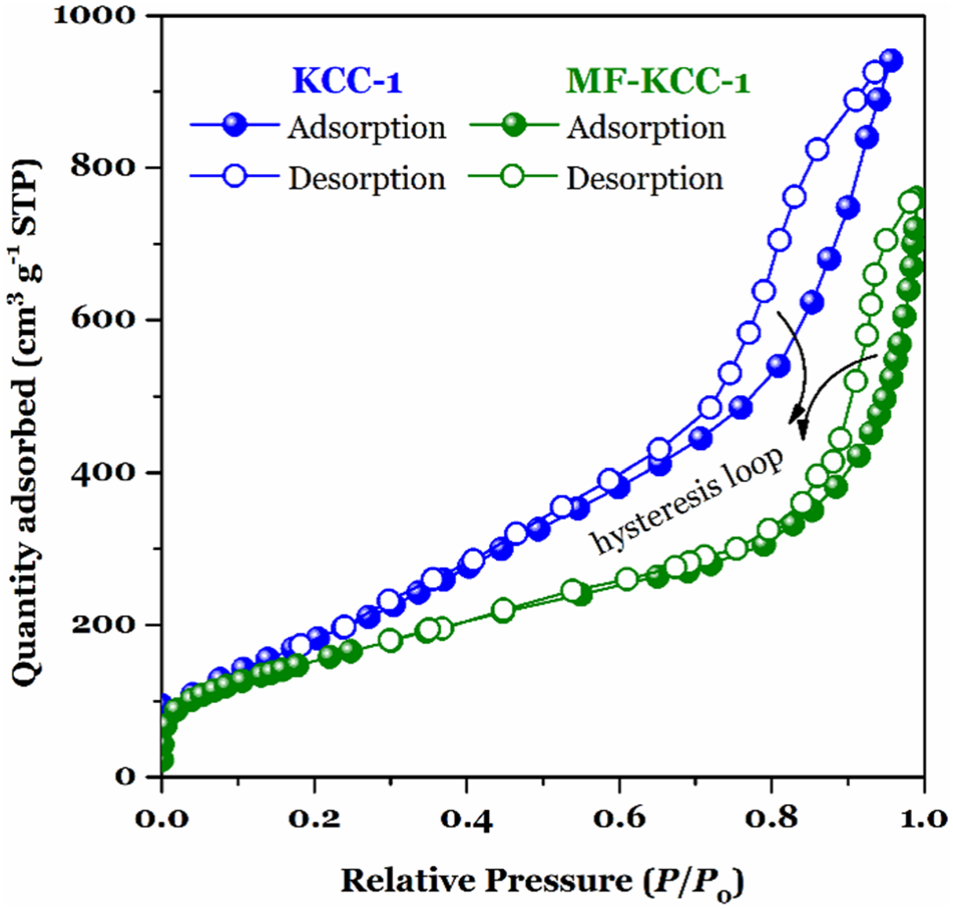
The N_2_ adsorption–desorption isotherms of KCC-1 and MF-KCC-1.

The N_2_ adsorption–desorption isotherms of KCC-1 and MF-KCC-1 revealed a characteristic type IV curve with a typical H3 hysteresis loop (Fig. 5), which is consistent with literature reports on standard KCC-1^43,52^. As for pure KCC-1, the Brunauer–Emmett–Teller (BET) surface area, Langmuir surface area, total pore volume (TPV), and Barrett–Joyner–Halenda (BJH) average pore diameter are obtained as 725 m^2^ g^−1^, 751 m^2^ g^−1^, 1.35 cm^3^ g^−1^, and 3.52 nm respectively, whereas the corresponding parameters of MF-KCC-1 have decreased to 572 m^2^ g^−1^, 618 m^2^ g^−1^, 0.98 cm^3^ g^−1^, and 2.23 nm as shown in Table 1. This reduction in surface area, pore volume, and average pore diameter (APD) during the surface functionalization process with SCAs is an expected phenomenon due to the attachment of organic moieties on the surface of the fibers. However, even after surface functionalization, MF-KCC-1 possesses a large surface area and high pore volume that can make it a potential material for use in adsorption and catalysis applications.

**Table 1:** Textural properties of KCC-1 and MF-KCC-1.

Samples	*S*_BET_ (m^2^ g^−1^)	*S*_Langmuir_ (m^2^ g^−1^)	TPV (cm^3^ g ^−1^)	APD (nm)
KCC-1	725	751	1.35	3.52
MF-KCC-1	572	618	0.98	2.23

### Adsorption studies

#### The impact of pH and adsorbent dosage

**Figure 6:**
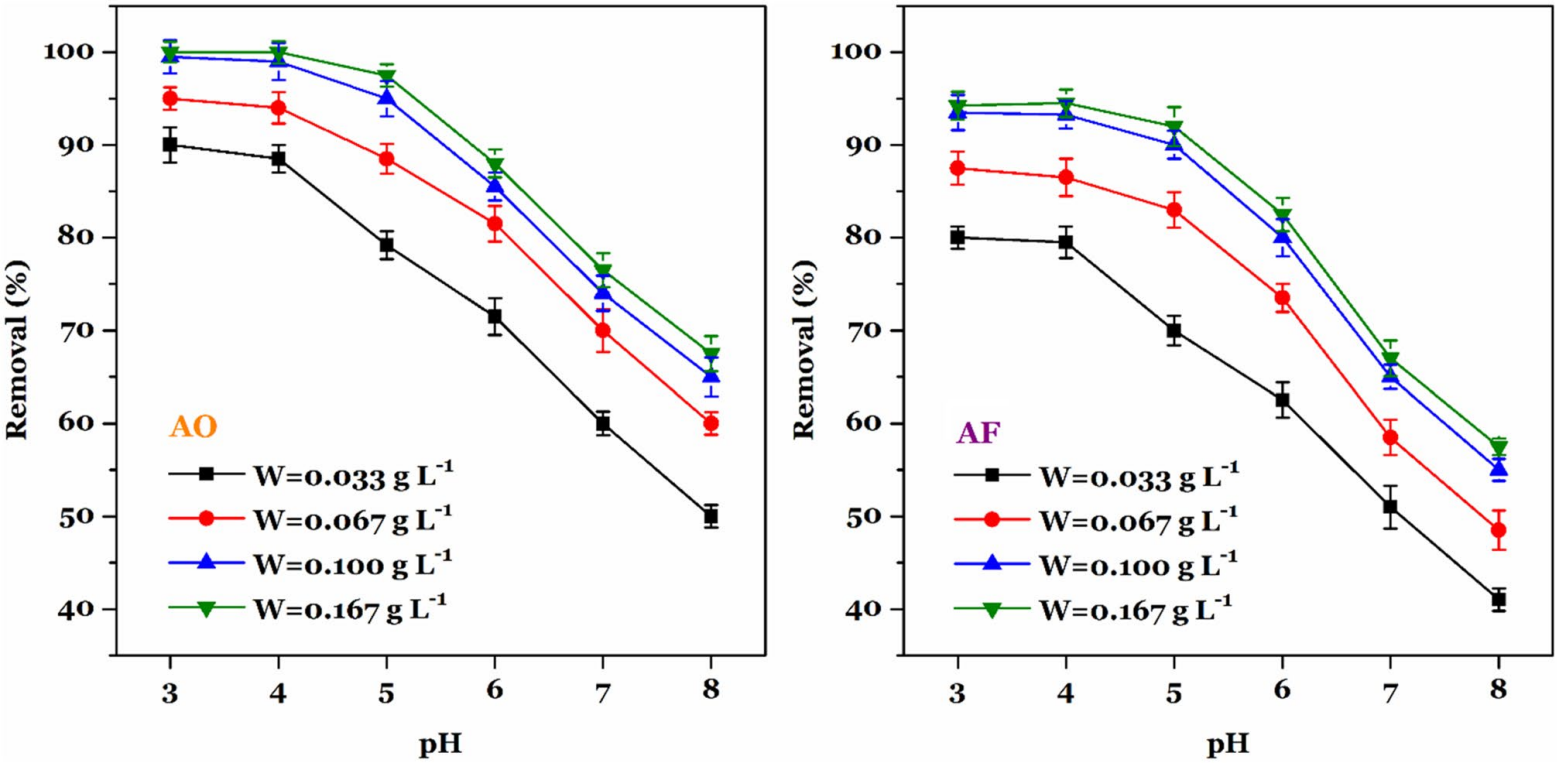
The effect of pH and adsorbent dosage on the removal percentage of AF (left) and AO (right) dyes ($C_{{\text{i}}}$ = 10 mg L^−1^, $t$ = 120 min, $T$ = 25 °C, shaking speed = 180 rpm).

The simultaneous effect of adsorbent dosage and pH on the adsorption of AF and AO was investigated. According to the adsorption data given in Fig. 6, as the amount of adsorbent increases from 0.033 g L^−1^ to 0.100 g L^−1^, the removal percentage increases continuously for both AF and AO dyes. With further increase in the adsorbent dosage, the removal percentage does not show a significant increase. Therefore, it can be concluded that for both AF and AO dyes, at a concentration of 10 mg L^−1^ and an adsorbent dosage of 0.100 g L^−1^ almost all adsorption sites are saturated. Also, for both AF and AO days, the highest removal percentages were observed at pH 3.0 to 4.0 in all adsorbent dosages. For AF and AO adsorption, the maximum uptake occurred at pH 3.0 and was up to 93.5% and 99.5%, respectively. As the pH of the solution increases, the removal percentage decreases steadily until at pH = 8.0 the removal percentages of AF and AO decrease to 55% and 66%, respectively. A similar trend has been reported in previous studies concerning adsorption of AF and AO by silica-based adsorbents^3,53^. At low pH, the surface of MF-KCC-1 became positively charged because of the protonation of functional groups. The positively charged surface of the MF-KCC-1 captures anionic AF and AO dyes in an aqueous medium through electrostatic attraction. As the pH of the solution increases, the surface charge density starts to decrease and the hydroxide ion concentration increases simultaneously, resulting in a decrease in adsorption of dyes by the adsorbent according to the following two main mechanisms^20^:

1. A decrease in the attractive electrostatic interactions between the surface of MF-KCC-1 and the anionic dye molecules due to electrostatic repulsion between the negatively charged surface of the adsorbent and anionic dyes.

2. The competitive behavior between hydroxide ions and anionic molecules for available adsorption sites.

Accordingly, the adsorbent dosage of 0.100 g L^−1^ and pH = 3.0 were chosen as optimal adsorbent dosage and solution pH for further investigations.

#### The impact of initial dye concentration and isotherm studies

**Figure 7:**
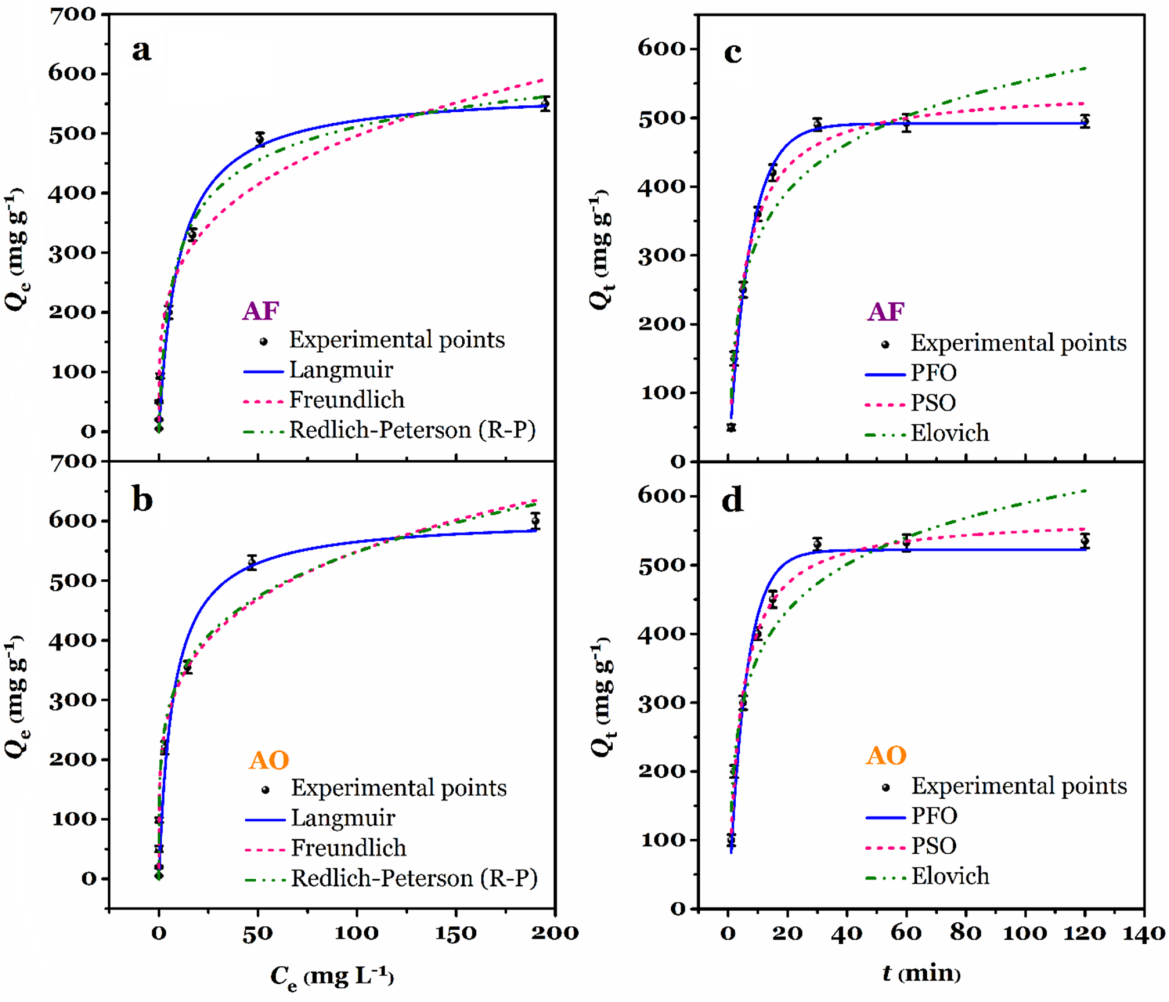
The effect of initial dye concentration on the adsorption capacity of AF (**a**) and AO (**b**) dyes and corresponding isotherm curves (pH = 3.0, $W$ = 0.100 g L^−1^, $C_{{\text{i}}}$ = 0.5–250 mg L^−1^, $t$ = 120 min, $T$ = 25 °C, shaking speed = 180 rpm). The effect of time on the adsorption capacity of AF (**c**) and AO (**d**) dyes and corresponding kinetic curves (pH = 3.0, $W$ = 0.100 g L^−1^, $C_{{\text{i}}}$ = 100 mg L^−1^, $t$ = 1–120 min, $T$ = 25 °C, shaking speed = 180 rpm).

The effect of initial concentration on the adsorption capacity of AF and AO were studied and different nonlinear isotherm models, namely Langmuir (Eq. 4, Table 2), Freundlich (Eq. 5, Table 2), and Redlich–Peterson (R–P, Eq. 6, Table 2), were used to fit the experimental data. As shown in Fig. 7a and b, for both AF and AO dyes, an increase in the concentration causes the adsorption capacity to increase continually. When the initial concentration lies between 0.5 and 100 mg L^−1^, the adsorption capacity to increases with a sharp slope. However, at initial concentrations ($C_{{\text{i}}}$) above 100 mg L^−1^, the adsorption slope decreases until it reaches an almost constant value at an initial concentration of 250 mg L^−1^. The maximum experimental adsorption capacity ($Q_{{{\text{m}},{\text{exp}}.}}$) for AF and AO were 550.5 and 600.0 mg g^−1^, respectively. To better understand the adsorption isotherms involved in the removal process of AF and AO by MF-KCC-1 from aqueous media, Langmuir, Freundlich, and R–P isotherm models were used and the relevant parameters after nonlinear regression analysis were given in Table 2. For AF adsorption, the obtained $R^{2}$ values, after nonlinear fitting of isotherm models, are in the following order: 0.9731 for Langmuir, 0.9713 for R–P, and 0.9547 for Freundlich. For AO adsorption, the $R^{2}$ values are in the following order: 0.9707 for Freundlich, 0.9672 for R–P, and 0.9527 for Langmuir. Although the $R^{2}$ values obtained for both Langmuir and Freundlich models are high, other isotherm parameters must be considered to determine which model is more consistent with the experimental data.

**Table 2:** Nonlinear forms of isotherms and kinetics equations and corresponding parameters and values.

Models	Equations	Parameters^a^	Values	
			AO	AF
Isotherms		$Q_{{{\text{m}},{\text{exp}}.}}$(mg g^−1^)	600.0	550.5
(4)				
Langmuir	$Q_{{\text{e}}} = \frac{{Q_{{{\text{m}},{\text{cal}}.}} \cdot K_{{\text{L}}} \cdot t}}{{1 + K_{{\text{L}}} \cdot C_{{\text{e}}} }}$	$Q_{{{\text{m}},{\text{cal}}.}}$(mg g^−1^)	605.9	574.5
		$K_{{\text{L}}}$(L mg^−1^)	0.1388	0.0991
		$R^{2}$	0.9527	0.9731
(5)				
Freundlich	$Q_{{\text{e}}} = K_{{\text{F}}} \cdot C_{{\text{e}}}^{1/n}$	$K_{{\text{F}}}$((mg g^−1^) (L mg^−1^)^1/n^)	193.8	149.7
		$n$(–)	4.422	3.841
		$R^{2}$	0.9707	0.9547
(6)				
R–P	$Q_{{\text{e}}} = \frac{{K_{{{\text{RP}}}} \cdot C_{{\text{e}}} }}{{1 + \alpha_{{{\text{RP}}}} \cdot C_{{\text{e}}}^{g} }}$	$K_{{{\text{RP}}}}$(L g^−1^)	3210.2	92.58
		$\alpha_{{{\text{RP}}}}$(mg L^−1^) ^–*g*^	15.409	0.2734
		$g$($0 < g < 1$)	0.7893	0.8978
		$R^{2}$	0.9672	0.9713
Kinetics		$Q_{{{\text{e}},{\text{exp}}.}}$(mg g^−1^)	535.2	495.1
(7)				
PFO	$Q_{{\text{t}}} = Q_{{{\text{e}},{\text{cal}}.}} \cdot \left( {1 - e^{{ - k_{1} \cdot t}} } \right)$	$Q_{{{\text{e}},{\text{cal}}.}}$(mg g^−1^)	521.8	491.8
		$k_{1}$(min^−1^)	0.1711	0.1394
		$R^{2}$	0.9704	0.9921
(8)				
PSO	$Q_{{\text{e}}} = \frac{{Q_{{{\text{e}},{\text{cal}}.}}^{2} \cdot k_{2} \cdot t}}{{1 + Q_{{{\text{e}},{\text{cal}}.}} \cdot k_{2} \cdot t}}$	$Q_{{{\text{e}},{\text{cal}}.}}$(mg g^−1^)	571.5	544.5
		$k_{2} \times 10^{ - 4}$(g mg^−1^ min^−1^)	4.22	3.41
		$R^{2}$	0.9904	0.9801
(9)				
Elovich	$Q_{{\text{t}}} = \frac{1}{\beta } \cdot \ln \left( {\alpha \cdot \beta } \right) \cdot t$	$\alpha$(mg g^−1^ min^−1^)	424.1	255.4
		$\beta \times 10^{2}$(g mg^−1^)	1.03	1.01
		$R^{2}$	0.9174	0.9103

^*a*^ $Q_{{{\text{m}},{\text{exp}}.}}$: experimental maximum adsorption capacity; $Q_{{{\text{m}},{\text{cal}}.}}$: calculated maximum adsorption capacity; $K_{{\text{L}}}$: Langmuir isotherm constant; $K_{{\text{F}}}$: Freundlich isotherm constant; $n$: Freundlich isotherm constant; $K_{{{\text{RP}}}}$ and $\alpha_{{{\text{RP}}}}$ are R-P isotherm constant; $g$ is R-P isotherm constant;$Q_{{{\text{e}},{\text{exp}}.}}$: experimental adsorption capacity at equilibrium; $Q_{{{\text{e}},{\text{cal}}.}}$: calculated adsorption capacity; $k_{1}$: PFO rate constant; $k_{2}$: PSO rate constant; $\alpha$ and $\beta$ are Elovich kinetic consatnts.

As shown in Table 2, for both AF and AO, the calculated maximum adsorption capacity ($Q_{{{\text{m}},{\text{cal}}.}}$ = 574.5 mg g^−1^ for AF and $Q_{{{\text{m}},{\text{cal}}.}}$ = 605.9 mg g^−1^ for AO) obtained by the Langmuir model is close to the corresponding experimental value ($Q_{{{\text{m}},{\text{exp}}.}}$ = 550.5 mg g^−1^ for AF and $Q_{{{\text{m}},{\text{exp}}.}}$ = 600.0 mg g^−1^ for AO), indicating the good agreement of this model with the experimental data. Also, according to the literature review, in the R-P equation, the closer $g$ parameter is to one and zero, the closer the experimental data are to the Langmuir and Freundlich models, respectively (for $g$ = 1 and $g$ = 0 the R-P model becomes the Langmuir and the Freundlich model, respectively)^25,46,49^. The obtained $g$ values for adsorption of AF and AO were found to be 0.8978 and 0.7893 which is indicative of a closer correlation of the adsorption isotherm data of both dyes with the Langmuir model.

Based on the Langmuir model, it can be assumed that AF and AO anionic dyes adsorb on a monomolecular layer of the MF-KCC-1 with unique localized adsorption sites without any special interaction between these dyes^54^.

#### The impact of contact time and kinetic studies

The impact of contact time in the adsorption process is of great economic importance. Accordingly, the effect of contact time on the adsorption of AF and AO by MF-KCC-1 was monitored and the obtained results are given in Fig. 7c and d. The amount of adsorption capacity for both AF and AO reached its maximum in the first 30 min and then reaches equilibrium. Consequently, in the removal process of AF and AO, 30 min contact time is the optimal time to reach adsorption equilibrium. In order to investigate the adsorption mechanisms involved in the adsorption process of AF and AO by MF-KCC-1, three different kinetic adsorption models, including pseudo-first-order (PFO), pseudo-second-order (PSO), and Elovich, were used, and corresponding computational data and $R^{2}$ values after nonlinear fitting are given in Table 2. According to the data in Table 2, the PFO and PSO kinetic models have a higher $R^{2}$ values than the Elovich kinetic model, which indicates that PFO and PSO models are more consistent with the experimental adsorption data. The $R^{2}$ trend for kinetic models is as follows: for adsorption of AF: PFO ($R^{2}$ = 0.9921) > PSO ($R^{2}$ = 0.9801) > Elovich ($R^{2}$ = 0.9103); and for adsorption of AO: PSO ($R^{2}$ = 0.9904) > PFO ($R^{2}$ = 0.9704) > Elovich ($R^{2}$ = 0.9174). Comparing the $R^{2}$ values for the PFO and PSO models, it is clear that the PFO and PSO models have higher $R^{2}$ values for AF and AO, respectively. However, for the adsorption of both dyes, the equilibrium adsorption capacities ($Q_{{{\text{e}},{\text{exp}}.}}$ = 495.1 mg g^−1^ for AF and 535.2 mg g^−1^ for AO) are more in line with the theoretical adsorption capacities ($Q_{{{\text{e}},{\text{cal}}.}}$ = 491.8 mg g^−1^ for AF and $Q_{{{\text{e}},{\text{cal}}.}}$ = 521.8 mg g^−1^ for AO) obtained from the PFO model, indicating that the PFO model is more consistent with the experimental data than the PSO model. As a result, the adsorption kinetics of AF and AO are a combination of both PFO and PSO kinetic models, in which the PFO model (fast adsorption response) plays a more significant role.

#### Comparison study

The MF-KCC-1 showed a remarkable adsorption performance for both AF and AO compared with other adsorbents in terms of both adsorption capacity and adsorption time. According to Table 3, for removal of AO from aqueous solution, only the adsorbent prepared by Soltani and his colleagues (LDH/MOF HNC, 2020)^18^ shows both higher adsorption capacity and shorter adsorption time compared to MF-KCC-1 adsorbent, and the other adsorbents show lower adsorption performance than MF-KCC-1. This excellent adsorption performance may be due to the unique fibrous structure of the MF-KCC-1 which facilitates easier access to abundant surface adsorption sites. Also, many functional organic groups like –SH, –S–S–S–S–, and –NH_2_ grafted on the surface of silica fibers increase attractive interactions with the AF and AO dye molecules.

**Table 3:** Maximum adsorption capacities for AF and AO by various adsorbents (NR: not reported; DDW: double distilled water; RT: room temperature).

Adsorbents	Year	$Q_{{{\text{m}},{\text{cal}}.}}$(mg g^−1^)		Conditions			Ref
		AO	AF	pH	*t* (min)	$T$(°C)	
MF-KCC-1	2020	605.9	574.5	3.0	30	25	This work
NH_2_-MIL-101(Cr)@Au	2020	419.85	–	5.0	30	RT	^55^
LDH/MOF HNC	2020	1173	–	7.0	15	20	^18^
de-oiled biomass	2019	–	9.9	DDW	50	35	^8^
γ-Fe_2_O_3_@C@UiO-66-NH_2_	2019	48.12	31.54	NR	180	25	^56^
CMC/BC	2018	–	253.2	2.0	60	20	^57^
MnO_2_/MCM-41	2015	909.99	716.17	2.0	150	20	^3^
HCZ	2014	38.96	-	1.0	60	30	^58^
NH_2_-MCM-41	2014	278.38	140.60	2.0–3.0	240	25	^53^
CMC	2013	–	105.71	5.0	120	25	^59^

Biomass: *Sargassum myriocystum*; CMC/BC: carboxymethyl-chitosan/bentonite composite; CMC: chemically modified cellulose; HCZ: hexadecyltrimethylammonium bromide coated zeolite.

## Conclusion

In summary, we have synthesized a multi-functionalized fibrous silica KCC-1 bearing amine (–NH_2_), mercapto (–SH), and tetrasulfide (–S–S–S–S–) functional groups. Pure KCC-1 was prepared based on a conventional sol–gel-hydrothermal method and then functionalized via a simple post-grafting approach to yield MF-KCC-1. FESEM and TEM images revealed that both KCC-1 and MF-KCC-1 particles possess a wrinkled spherical morphology as well as uniform fibrous structure. BET model revealed that KCC-1 and MF-KCC-1 have a high surface area of 725 m^2^ g^−1^ and 572 m^2^ g^−1^, respectively, with mesoporous structure. Due to its high surface area, abundant active surface groups, and unique fibrous structure, MF-KCC-1 was used as an adsorbent to remove AF and AO anionic dyes from aqueous media. The impact of important adsorption factors, such as pH, adsorbent dosage, initial dye concentration, and contact time, on the removal process were investigated and optimal conditions were obtained. In order to gain a better understanding of plausible adsorption mechanisms involved in the adsorption process, isotherm and kinetic studies were conducted and it was found that among the different isotherm and kinetic models used for both AF and AO dyes the Langmuir isotherm model and the PFO kinetic model show the most agreement with the experimental data. The calculated maximum adsorption capacity for AF and AO, according to the Langmuir model, was found to be 574.5 mg g^−1^ and 605.9 mg g^−1^, respectively, surpassing most adsorption capacities reported until now. We believe that the described fabrication method and adsorbent design in this study can inspire the synthesis and development of new multi-functionalized KCC-1 nanoparticles for use as adsorbents in environmental applications like adsorption, extraction, and even catalysis.

## Material and methods

### Chemicals

Tetraethyl orthosilicate (TEOS, ≥ 99%), cetyltrimethylammonium bromide (CTAB, ≥ 99%), (3-aminopropyl)triethoxysilane (APTES, 99%), bis[3-(triethoxysilyl)propyl] tetrasulfide (TESPTS, ≥ 90%), (3-mercaptopropyl)triethoxysilane (MPTES, ≥ 80%), acid Fuchsin (AF, dye content 70%), acid orange II (AO, ≥ 98%) were purchased from Sigma-Aldrich (Germany). Cyclohexane (≥ 99.9%), hydrochloric acid (HCl, 37%), urea (≥ 99%), n-amyl alcohol (≥ 98.5), and sodium hydroxide (NaOH, pellets, ≥ 97%) were purchased from Merck Millipore (Germany). Ethanol (96% and absolute) and acetone (HPLC grade) were purchased from Mojallali Chemical Co. (Tehran, Iran).

### Synthesis of KCC-1 and MF-KCC-1

Pure KCC-1 was fabricated according to a typical sol–gel-hydrothermal method (in a Teflon-lined stainless steel autoclave) reported by Soltani and co-workers^20,47^. In a typical synthesis route, in a 1-L Teflon cylinder, urea (3.6 g, 59.9 mmol) and CTAB (3 g, 8.23 mmol) were first dissolved in 250 mL pure water under stirring for 15 min at 25 °C. To the above solution, a mixture of TEOS (15 mL, 72 mmol) and cyclohexane (250 mL) was added. The mixture was further stirred for 15 min and then n-amyl alcohol (18 mL) was added. The mixture was stirred for 20 min before placing the Teflon container in a stainless-steel autoclave. The autoclave was then transferred to an electric oven and kept at 120° C for 6 h. At the end of the reaction, the autoclave was allowed to cool to room temperature. The white gel-like product was separated by centrifugation and washed several times with ethanol and water, followed by oven-drying (60 °C, overnight) and calcination (550 °C, 6 h) in the air to yield fine white powders of pure KCC-1.

KCC-1 was functionalized with SCAs, namely APTES, MPTES, and TESPTS, via the following post-modification technique. In a typical procedure, 3 g KCC-1 and 300 mL ethanol were added into a 1-L round bottom flask and ultrasonicated for 15 min. Afterward, 1.5 mL of SCAs mixture (molar ratio APTES/MPTES/TESPTS = 1:1:1) was added to the flask and ultrasonicated for a further 15 min, followed by refluxing for 24 h. After cooling to room temperature, the obtained white product was centrifuged, rinsed repeatedly with ethanol and water, and oven-dried (60 °C for 24 h) to yield MF-KCC-1.

### Instruments and characterization of samples

In order to investigate the qualitative characterization of functional groups of samples and also to find out whether SCAs had been grafted on KCC-1 successfully, Fourier Transform Infrared (FTIR, Avatar 370, Thermo Nicolet, USA) spectra of the samples were recorded from 4000 to 400 cm^−1^ wavenumber.

A field emission scanning electron microscope (FESEM, MIRA3 TESCAN-XMU, Kohoutovice, Czech Republic) was applied to observe the surface morphology of KCC-1 and MF-KCC-1 before and after grafting of SCAs on the surface of KCC-1. An energy-dispersive X-ray (EDX) spectrometer was used to observe the distribution of the elements on the surface of KCC-1.

The fibrous structure of the samples was visualized by a transmission electron microscope (TEM, Philips CM120) with a tension voltage of 120 kV.

To measure the porosity and adsorption behavior of the KCC-1 and MF-KCC-1, a volumetric N_2_ adsorption–desorption apparatus (BELSORP-mini II, Osaka, Japan) was used. The surface area of the samples was calculated according to the Brunauer–Emmett–Teller (BET) and Langmuir models. the Barrett–Joyner–Halenda (BJH) method was used to measure the pore volume and pore sized distribution of the samples.

The concentrations of AF and AO in the aqueous solutions were measured using a Spectrophotometer (Model, UV-1201, Shimadzu, Tokyo, Japan) at λ_max._ = 524 nm and λ_max_ = 486 nm, respectively.

### Adsorption experiments

The removal percentage and adsorption capacities at equilibrium ($Q_{{\text{e}}}$, mg g^−1^) and any time $t$ ($Q_{{\text{t}}}$, mg g^−1^) were calculated using the following equations:

1 $$ \% Removal = \frac{{C_{{\text{i}}} - C_{{\text{e}}} }}{{C_{{\text{i}}} }} \times 100 $$

2 $$ Q_{{\text{e}}} = \left( {C_{{\text{i}}} - C_{{\text{e}}} } \right) \times \frac{V}{W} $$

3 $$ Q_{{\text{t}}} = \left( {C_{{\text{i}}} - C_{{\text{t}}} } \right) \times \frac{V}{W} $$

where, $C_{{\text{i}}}$ (mg L^−1^), $C_{{\text{e}}}$ (mg L^−1^), and $C_{{\text{t}}}$ (mg L^−1^) are initial concentration, equilibrium concentration, and concentration at any time $t$, respectively. $V$ (mL) and $W$ (g L^−1^) represent the volume of solution and the amount of adsorbent, respectively.

The simultaneous effect of pH and adsorbent dosage ($W$, g L^−1^) was investigated by adding a certain amount of MF-KCC-1 ($W$ = 0.033, 0.067, 0.100, and 0.167 g L^−1^) into 100-mL polypropylene bottles containing 30 mL AF and AO severally. The bottles were shaken using an IKA KS 3000ic control incubator shaker (Germany) at 180 rpm min^−1^ for 120 min at 25 °C. Initial concentrations of both dyes were 10 mg L^−1^. After shaking, the samples were centrifuged and the residual concentrations of each dye in the solutions were measured by UV spectrophotometer.

Moreover, the impact of the initial concentration of dye on the adsorption performance was investigated by the same procedure and diluting stock solutions (1000 mg L^−1^) of AF and AO into 0.5, 2, 5, 10, 25, 50, 100, and 250 mg L^−1^ (pH = 3.0, $V$ = 30 mL, $W$ = 0.100 g L^−1^, $t$ = 120 min, $T$ = 25 °C, shaking speed = 180 rpm min^−1^). In a similar way, the effect of contact time on the adsorption was conducted by measuring the concentration of samples at 1, 2, 5, 10, 15, 30, 60, and 120 min contact time ($C_{{\text{i}}}$ = 100 mg L^−1^, pH = 3.0, $V$ = 30 mL, $W$ = 0.100 g L^−1^, $T$ = 25 °C, shaking speed = 180 rpm min^−1^).
